# Supplementary material for: Potential high-frequency off-target mutagenesis induced by CRISPR/Cas9 in Arabidopsis and its prevention
Source: Plant Mol Biol. 2018 Feb 23;96(4):445–56. doi: 10.1007/s11103-018-0709-x (PMC5978904; doi:10.1007/s11103-018-0709-x)
Supplement: Supplementary file 1 — Supplementary material 1 (PDF 7983 KB) [file 11103_2018_709_MOESM1_ESM.pdf]

Supplementary material

Table of contents

Fig. S1 2
Fig. S2 3
Fig. S3 4
Fig. S4 5
Fig. S5 6
Table S1 7
Table S2 8
Table S3 9
Table S4 10
Table S5 11
Table S6 12
Table S7 13
Table S8 15
Table S9 16
Table S10 17
Table S11 18
Methods S1 19
Appendix S1–S5 23

Fig. S1

(A)

sgR-ETC2 (in pEa/b)

tsgR(o/m)-ETC2-2 (in pEc/d)

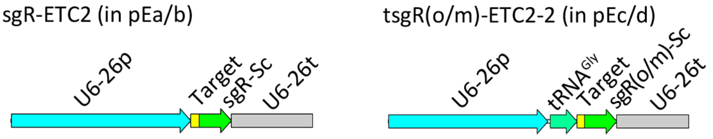

(B)

Cas9 driven by EC1.2 promoter or EC1.2-EC1.1 fusion promoter (EC1fp)

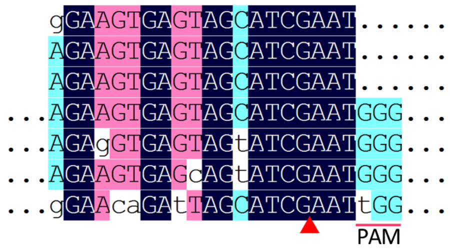

Vector	pEa	pEb	pEc	pEd
EC1.2p:Cas9	+	–	–	–
EC1fp:Cas9	–	+	+	+
tRNA	–	–	+	+
sgR-ETC2	+	+	–	–
tsgR(o)-ETC2-2	–	–	+	–
tsgR(m)-ETC2-2	–	–	–	+
ETC2 (on-T)	13.4% (15/112)	12.0% (6/50)	5.2% (5/96)	9.2% (9/98)
CPC (off-T)	13.3% (2/15)	16.7% (1/6)	20.0% (1/5)	66.7% (6/9)
TRY (off-T)	0	0	0	0
AT5G50230 (off-T)	0	0	0	0

Fig. S1 Strategy of tRNA-sgRNA(m) fusion conferred much higher efficiency of mutations in the *ETC2* gene than that with tRNA-sgRNA(o) fusion. **a** Physical maps of three types of sgRNA expression cassettes. Sc, scaffold; sgR(o/m), sgRNA with original/mutant scaffold. **b** On-target and off-target mutation frequencies in T1 transgenic lines harboring one of the three types of sgRNA expression cassettes and one of the two types of Cas9 expression cassettes. The names of the four CRISPR/Cas9 binary vectors and their Cas9/sgRNA expression cassettes are indicated on the upper right. The aligned sequences of the three sgRNAs, on/off-target genes are displayed on the left. The mutation frequencies are indicated on the right of the target sites. The on-target mutation efficiency was calculated based on the percent ratio of the number of mutants to the total number of T1 plants. The off-target mutation frequency was calculated based on the ratio of the number of mutants harboring off-target mutations to the total number of mutant plants. Only aligned regions of interest are displayed.

Fig. S2

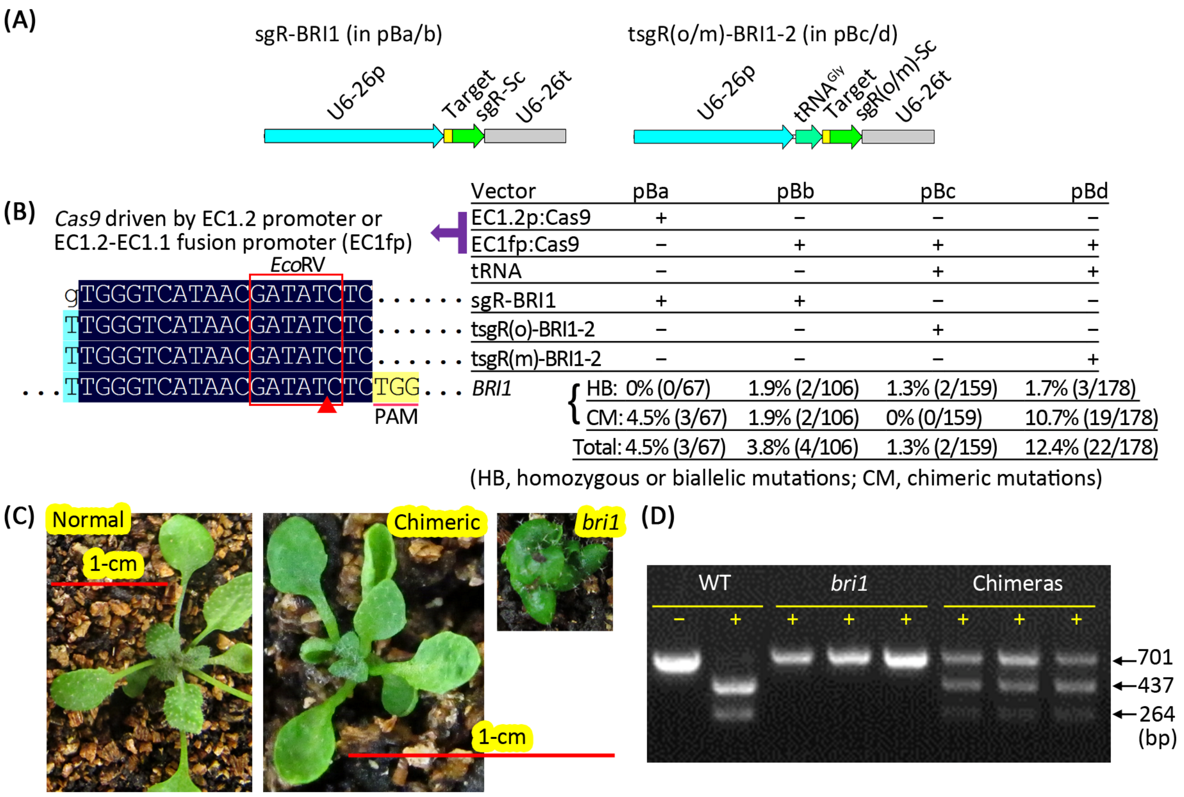

Fig. S2 Strategy of tRNA-sgRNA(m) fusion confers significantly higher efficiency of mutations in the *BRI1* gene than that with tRNA-sgRNA(o) fusion. **a** Physical maps of three types of sgRNA expression cassettes in four vectors. Sc, scaffold; sgR(o/m), sgRNA with original/mutant scaffold. **b** Frequencies of mutations in the *BRI1* gene of T1 transgenic lines harboring one of the three types of sgRNA expression cassettes and one of the two types of Cas9 expression cassettes. The names of the four CRISPR/Cas9 binary vectors and their Cas9/sgRNA expression cassettes are indicated on the upper right. The aligned sequences of the three sgRNAs and target genes are displayed on the left. The mutation frequencies are indicated on the right of the target sites. The on-target mutation efficiency was calculated based on the percent ratio of number of mutants to total number of T1 plants. Only aligned regions of interest are displayed. **c** Representative phenotypes of T1 pEd transgenic plants with chimeric or non-chimeric (homozygous or biallelic) mutations in the *BRI1* gene. **d** *Eco*RV digestion analysis of PCR products amplified from the chimeric or non-chimeric mutant plants. −/+ indicate digestion with or without *Eco*RV, respectively. Only results from three representative T1 pEd transgenic plants for each of the two types of mutations are displayed.

Fig. S3

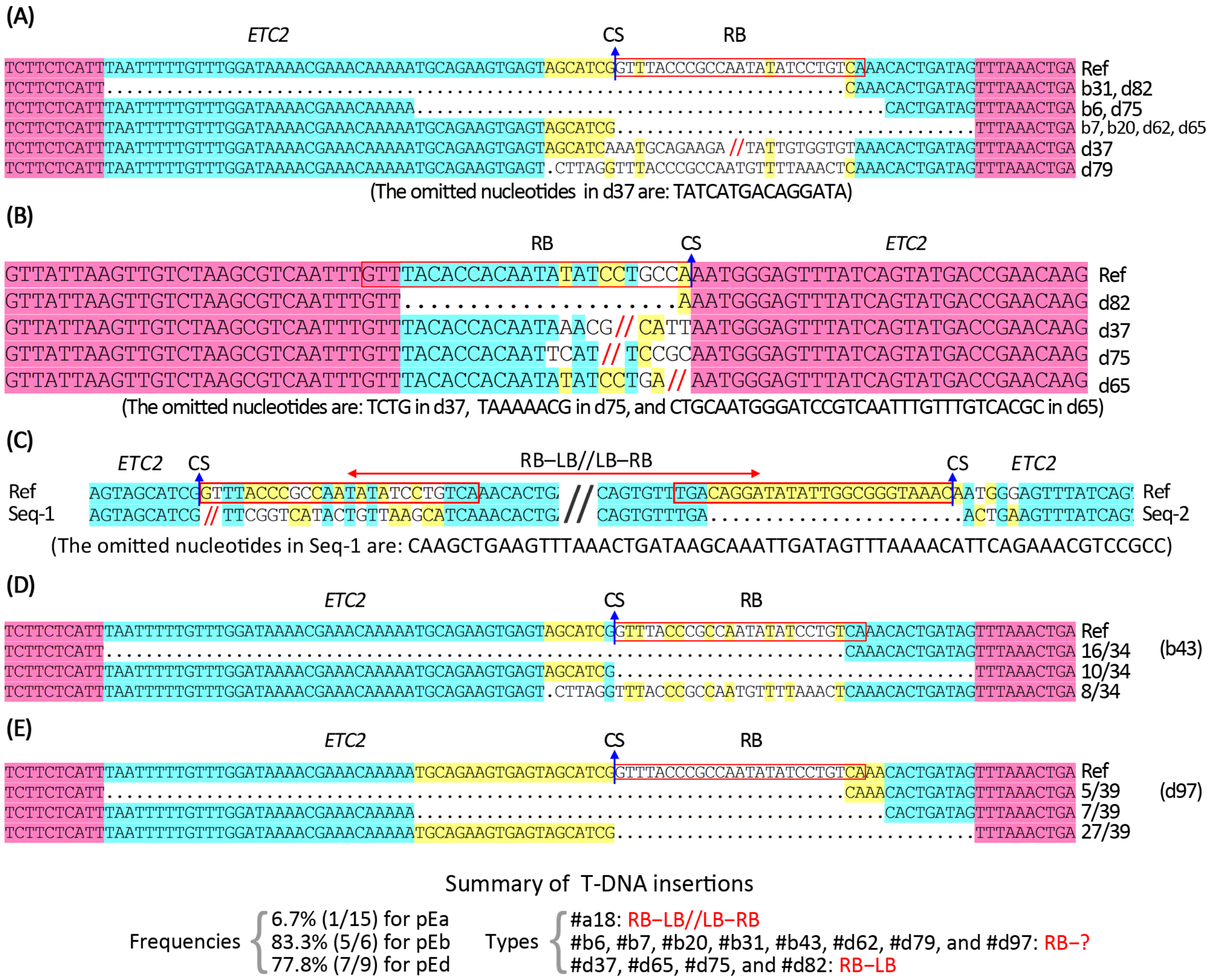

Fig. S3 Juncture sequences between T-DNA and *ETC2* sequences before or behind the cleavage site of CRISPR/Cas9. Alignment of actual juncture sequences from direct sequencing of PCR products (**a**–**c**) or sequencing of cloned PCR products (**d**, **e**) with predicted *ETC2* and T-DNA sequences. Only aligned regions of interest are displayed. RB or LB sequences are boxed. For cloned PCR products, the ratios of number of clones with the same sequences to total number of sequenced clones are indicated. CS, cleavage site. Ref, reference sequence of the junctures. Seq, actual sequence from sequencing. Dots represent deletions. Summary of T-DNA insertions including the frequencies of T-DNA insertions into mutant plants transformed with different binary vectors (pTa, pTb, and pTd), and types of T-DNA insertions, are indicated. Question marks indicate the regions where the T-DNA border could not be detected.

Fig. S4

(A)

```
                 HAB1                    CS      RB
TTATGAACTAGATTGTATACCGCTTTGGGGCACGGTTTCCATTGTTTACCCGCCAATATATCCTGTCAAACACTGATAGTTTAAACTGAAGGCGGGAAACGACAATCTGATC Ref
TTATGAACTA.......................................TCAGCTTGAGCTTGGATCAAACACTGATAGTTTAAACTGAAGGCGGGAAACGACAATCTGATC #1
TTATGAACTAGATTGTATACCGCTTTGGGGCACGGTTTCCATT..............................GACGGGTAAACAAATAGATTTTTATTAGTCAATCTGATC #4
TTATGAACTAGATTGTATACCGCTTTGGGGCACGGTTTCCATT.......................CAAACACTGATAGTTTAAACTGAAGGCGGGAAACGACAATCTGATC #14
```

(B)

```
                      RB             CS                      HAB1
CCGATCGCCCTTC//GTTTGACAGGATATATTGGCGGGTAAACCAAGGGAATA//TCTGAAACTACCCATCAAAATGCTTATGGGGACCATGA Ref
CCGATCGCCC..............................................AAAAAAAAGCACCGACTCGGGACCATGA #1
```

For T-DNA: // = CCAACAGTTGCGCAGCCTGAATGGCGAATGCTAGAGCAGCTTGAGCTTGGATCAGATTGTCGTTTCCCGCCTTCAGTTTAAACTATCAGT
For HAB1: // = GATCTGAGATGGAGGATGCTTTTGCCGTGTCACCTCATTT

Summary of T-DNA insertions:
- #1: RB–LB//LB–RB
- #4: RB–?
- #14: RB–?

Fig. S4 Juncture sequences between T-DNA and *HAB1* before or behind the cleavage site of CRISPR/Cas9. **a**, **b** Alignment of actual juncture sequences from direct sequencing of PCR products with predicted *HAB1* and T-DNA sequences. Only aligned regions of interest are displayed. RB or LB sequences are boxed. CS, cleavage site. Ref, reference sequence of the junctures. Seq, actual sequence from sequencing. Dots represent deletions or absence. Summary of T-DNA insertions into different lines is indicated. Question marks indicate the regions where the T-DNA border could not be detected.

Fig. S5

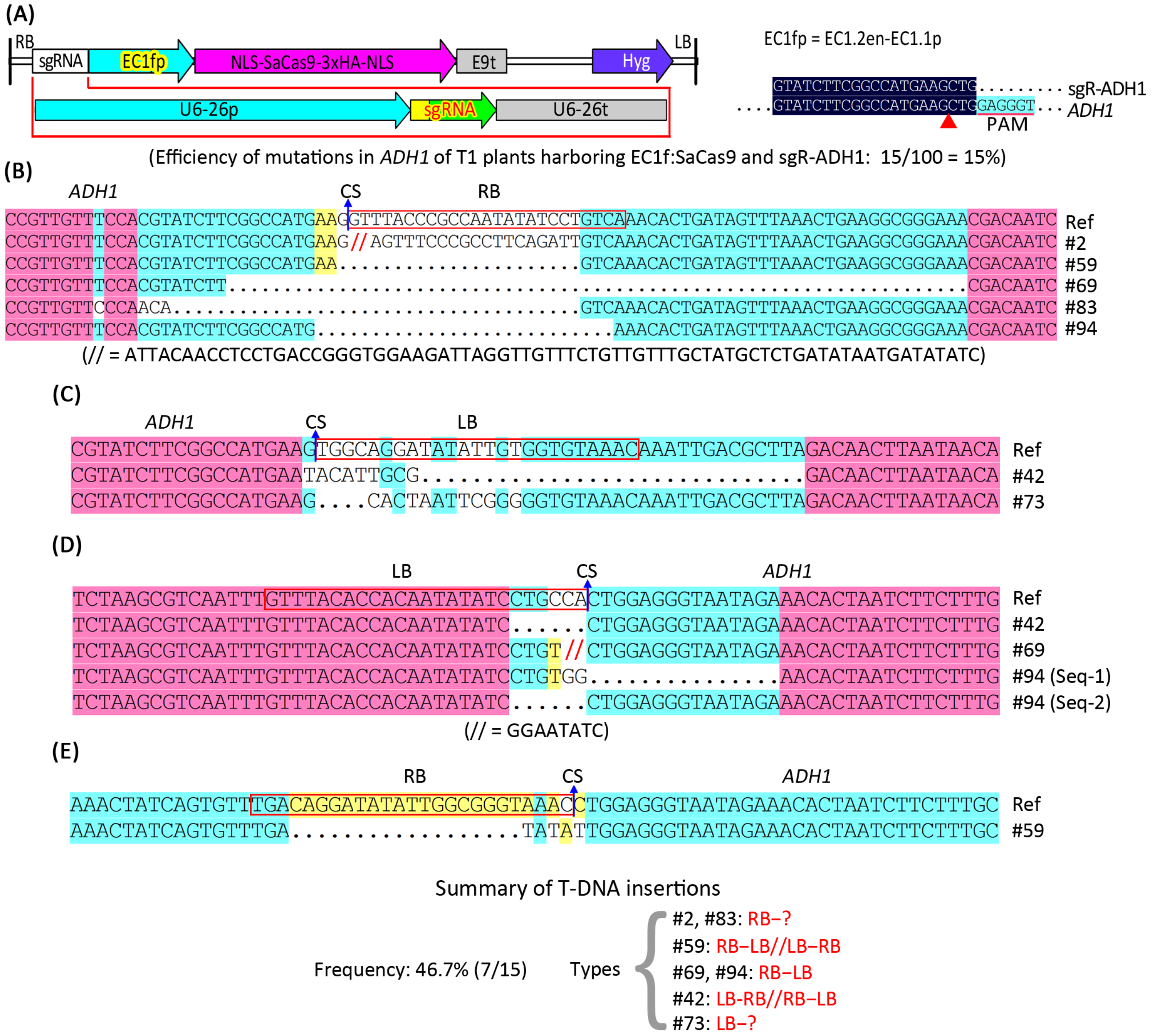

Fig. S5 Juncture sequences between T-DNA and *ADH1* before or behind the cleavage site of CRISPR/SaCas9. **a** Physical map of T-DNA harboring maize-codon optimized SaCas9 and sgRNA targeting the *ADH1* gene. Alignment of sgRNA with its target gene is indicated. Only aligned regions of interest are displayed. Efficiency of mutations in *ADH1* of T1 plants harboring EC1f:SaCas9 and sgR-ADH1 is indicated and calculated by the ratio of the number of *adh1* mutants to the total number of T1 plants. **b**–**e** Alignment of actual juncture sequences from direct sequencing of PCR products, or sequencing of cloned PCR products, with predicted *ADH1* and T-DNA sequences. Only aligned regions of interest are displayed. Two types of juncture sequences (Seq-1/2) between T-DNA and CS-*ADH1* in line #94 are determined by sequencing of cloned PCR products. RB or LB is boxed. CS, cleavage site. Ref, reference sequence of the junctures. Seq, actual sequence from sequencing. Dots represent deletions or absence. Summary of T-DNA insertions into different lines is indicated. Question marks indicate the regions of the T-DNA border that could not be detected.

Table S1

Table S1 On-target and off-target mutation analysis of 15 T1 mutant plants

Line	ETC2	TRY		CPC	
		Off	On	Off	On
#1	+A/+T	0/0	+G/+G	0/0	+A/+T
#2	−24(×11)/+A(×6)	0/0	+G/+G	−629//others (*)	
#3	−4/−4	0/0	+A(×8)/−G(×7)	+A(×9)/+T(×8)	−G(×13)/+G(×7)
#4	+A/+A	+T/+T	+C/+C	+A/+A	+T−DNA (***)
#5	+T/+T	0(×12)/+A(×7)	+A(×11)/−19(×7)	+A/+A	+G/+G
#6	+A/+A	Inversion (**)		−629//others (*)	
#7	+A/+A	0/0	+G/+G	0/0	+T(×8)/−3+38(×11)
#8	−G(×9)/+T(×7)	0/0	+A/+A	−640//others (*)	
#9	−G(×10)/+C(×9)	0/0	−3(×6)//+G(×8) //+T(×5)	+A/+A	+C/+C
#10	−2(×7)/+A(×9)	0/0	+A/+A	0(×11)/+T(×6)	+G/+G
#11	+T/+T	0/0	−G/−G	−629/−629 (**)	
#12	+C/+C	0/0	−G(×13)/+G(×6)	0(×11)//+G(×7) //−2(×1)	−3(×10)/+G(×7)
#13	+T(×4)/−1+5(×8)	0/0	+T(×10)/−9(×8)	−629//others (*)	
#14	+C/+G	0/0	+T/+T	−629//others (**)	
#15	+T/+T	0/0	+T(×5)/−9(×5)	+A/+T	−3(×9)/+T(×8)

Off/On, off/on-target mutations. "+" indicates insertion, "−" indicates deletion, "0" indicates no mutation (wild-type allele). "BC" indicates biallelic or chimeric mutations not determined. The number following "+" or "−" indicates the number of bases inserted or deleted; if the number is 1, it was replaced with a specific base. Mutations were detected by direct sequencing of PCR products or sequencing of cloned PCR products. Two types of mutations from direct sequencing of PCR products were obtained based on double-peaks on a chromatograph. When mutations were detected by sequencing of cloned PCR products, the number of the same type of mutation is indicated in parentheses. Two alleles (in WT, homozygous or biallelic mutants, or heterozygous mutants) are separated by "/", whereas more than two alleles (in mosaic plants) are separated by "//" between two alleles. "Inversion" indicates the 629-bp fragment between the two cleavage sites of on-target and off-target sites was deleted and inversely inserted between the two cleavage sites. "−629" mutation type indicates that the 629-bp fragment was deleted, whereas "−640" mutation type indicates extra 11-bp at the two cleavage sites was deleted together with the 629-bp deletion. "Others" indicate chimeric mutations not determined. "*" indicates difficulty in original PCR amplifications, "**" indicates failure in original PCR amplifications, and "***" indicates failure in original and final PCR amplifications.

Table S2

Table S2 Frequencies of off-target mutations in T1 and T2 plants

pEb transgenic plants	T1	T2							
Original T1 line number	20*	48	6	31	20*	7	43	/	/
Renamed T1 line number	0	1	2	3	4	5	6	A	STDEV
Off-CPC	1/6	4/20	12/20	14/20	14/20	15/20	17/20	/	/
	16.7%	20.0%	60.0%	70.0%	70.0%	75.0%	85.0%	63.3%	22.7%
Off-T&C	0/50	0/264	29/319	0/232	4/347	9/278	15/177	/	/
	0.0%	0.0%	9.1%	0.0%	1.2%	3.2%	8.5%	3.7%	4.1%

The number of mutants and total number of T1 or T2 plants from which percentage was calculated are indicated as fractions. The efficiency of on-target mutations in the *ETC2* gene of T1 plants was 12.0% (6/50), and all the T2 plants were from the six T1 mutant lines. * The T1 line harboring chimeric off-target mutations in the *CPC* gene. Off/on, off/on-target mutations. T&C, *TRY* and *CPC*.

Table S3

Table S3 Efficiencies of mutations in *TRY* and *CPC* induced by mutant SpCas9 variants

	T1	T2							
		1	2	3	4	5	6	A	STDEV
Cas9	27/156	146/422	164/519	95/225	156/435	97/231	138/352	/	/
	17.3%	31.6%	34.6%	35.9%	39.2%	42.0%	42.2%	37.6%	4.3%
eCas9(1.0)	0/89	13/247	16/263	29/215	68/291	60/231	75/278	/	/
	0.00%	5.3%	6.1%	13.5%	23.4%	26.0%	27.0%	16.9%	9.9%
eCas9(1.1)	1/156	0/208	34/248	54/313	86/273	101/288	90/201	/	/
	0.6%	0.0%	13.7%	17.3%	31.5%	35.1%	44.8%	23.7%	16.4%
eCas9(1.2)	0/71	0/236	32/273	39/261	47/296	88/259	103/264	/	/
	0.0%	0.0%	11.7%	14.9%	15.9%	34.0%	39.0%	19.3%	14.6%
Cas9-HF1	0/67	0/213	1/277	1/223	2/204	4/264	17/298	/	/
	0.0%	0.0%	0.4%	0.4%	1.0%	1.5%	5.7%	1.5%	2.1%
HF1-e(1.1)	0/84	0/286	0/231	0/297	0/203	0/288	0/246	/	/
	0.0%	0.0%	0.0%	0.0%	0.0%	0.0%	0.0%	0.0%	0.0%
HF1a-e(1.1)	0/68	0/654	0/503	0/485	0/489	0/623	0/576	/	/
	0.0%	0.0%	0.0%	0.0%	0.0%	0.0%	0.0%	0.0%	0.0%
HF1b-e(1.2)	0/157	0/223	0/264	0/269	0/200	0/251	0/297	/	/
	0.0%	0.0%	0.0%	0.0%	0.0%	0.0%	0.0%	0.0%	0.0%
HF1a-e(1.2)	0/112	0/462	0/398	0/426	0/552	0/418	0/697	/	/
	0.0%	0.0%	0.0%	0.0%	0.0%	0.0%	0.0%	0.0%	0.0%

The number of mutants and the total number of T1 or T2 plants from which percentage was calculated are indicated as a fraction. See Table 1 for the mutations in SpCas9 variants.

Table S4

Table S4 Efficiencies of mutations in *BRI1* induced by mutant SpCas9 variants

	T1	T2							
		1	2	3	4	5	6	A	STDEV
Cas9	22/178	99/211	101/201	137/244	154/270	204/355	186/251	/	/
	12.4%	46.9%	50.2%	56.1%	57.0%	57.5%	74.1%	57.0%	4.7%
eCas9(1.1)	0/96	96/203	116/241	178/364	152/296	172/329	132/226	/	/
	0.0%	47.3%	48.1%	48.9%	51.4%	52.3%	58.4%	51.1%	4.1%
Cas9-HF1	0/50	0/243	0/200	1/225	2/258	2/214	4/207	/	/
	0.0%	0.0%	0.0%	0.4%	0.8%	0.9%	1.9%	0.7%	0.7%

The number of mutants and the total number of T1 or T2 plants from which percentage was calculated are indicated as a fraction.

Table S5

Table S5 Efficiencies of mutations in *ETC2* induced by mutant SpCas9 variants

	T1	T2							
		1	2	3	4	5	6	A	STDEV
Cas9	9/98	/	/	/	/	/	/	/	/
	9.2%	/	/	/	/	/	/	/	/
eCas9(1.1)	1/42	28/48	27/46	30/50	32/50	34/50	36/50	/	/
	2.4%	58.3%	58.7%	60.0%	64.0%	68.0%	72.0%	63.5%	5.6%
Cas9-HF1	0/50	18/50	19/49	20/50	21/49	22/49	27/50	/	/
	0.0%	36.0%	38.8%	40.0%	42.9%	44.9%	54.0%	42.8%	6.3%

The number of mutants and the total number of T2 plants from which percentage was calculated are indicated as a fraction.

Table S6

Table S6 Frequencies of off-target mutations induced by eSpCas9(1.1)

pE11-E transgenic plants	T2							
T1 line number	1	2	3	4	5	6	A	STDEV
On-target mutations<br>in the *ETC2* gene	28/48<br>58.3%	27/46<br>58.7%	30/50<br>60.0%	32/50<br>64.0%	34/50<br>68.0%	36/50<br>72.0%	/<br>63.5%	/<br>5.6%
Off-target mutations<br>in the *CPC* gene	0/28<br>0.0%	0/27<br>0.0%	0/30<br>0.0%	0/32<br>0.0%	0/34<br>0.0%	0/36<br>0.0%	/<br>0.0%	/<br>0.0%
Off-target mutations<br>in the *TRY* and *CPC* genes	0/899<br>0.0%	0/863<br>0.0%	0/1017<br>0.0%	0/978<br>0.0%	0/914<br>0.0%	0/1023<br>0.0%	/<br>0.0%	/<br>0.0%

The number of mutants and the total number of T2 plants from which percentage was calculated are indicated as a fraction.

Table S7

Table S7 Primers used in this study

Generation of sgRNA cassettes and Cas9 variants (5'→3')	
U6-BNF	atattacgtctcccatggcgacttgccttccgcacaatac
U6-BSR	aacatacgtctcactagttattggtttatctcatcg
Ap-BBF	attattgaagacatattgtgagaccttcggggaaatgtgcgc
Ap-BBR	attattgaagacttaaactgagacctgacgctcagtggaacg
sgRm-F	gaaacagcatagcaagttgaaataaggctagtccgt
sgRm-SaR	atttagagctcagcatagctctgaaactgagacctgacgctcagt
tRG-BbF	tgaagacatattgaacaaagcaccagtggtctagtg
tRG-R0	ttaccctgttatccctaggtctcatgcaccagccgggaat
tRG-BbR	tgaagactcaaactgagaccattaccctgttatcccta
Ap-BBF2	catagaagactatgcatgagaccttcggggaaatgtgcgc
Ap-BBR2	attattgaagacttaaactgagacctgacgctcagtggaacg
zCas9dF-F	acattatgccagtgaattggtctagaagatggctccgaag
zCas9dF-R	gaatcgtcaaccttcgccatctcgt
Construction of cloning CRISPR/Cas9 binary vectors (5'→3')	
mCherry-HMF	acatgcaagcttacaattgattgacaactaagctggcacaactatatttcc
mCherry-EAR	attacgaattcaacctaggctcaatacgataatttatttg
oAiCE-F	ctaggcgtaactataacggtcctaaggtagcg
oAiCE-R	aattcgctaccttaggaccgttatagttacgc
Generation of PCR template vectors (5'→3')	
Sp-XhF	atattactcgagaacccagtggacataagc
sgR-BbR	atacatttgaagacattgttgcaccgactcggtgccactt
tRG-BbF	ccatacattgaagacataacaaagcaccagtggtctagtg
Ap-XhR	attattatctcgagtgacgctcagtggaacg
tRM-BbF	ccatacattgaagacataacaacaaatcagagtggc
tRM-R	aatgaaccgtagcttgtgatgacac
sgRm-F2	gtttcagagctatgctggaaacagcatagcaagttgaa
sgRm-F0	gaaacagcatagcaagttgaaataaggctagtccgt
U629p-R	caatctcttagtcgactctaccaat
sgR-F	caccgttttagagctagaaatagcaagt
U629p-BbR	atttatttacgaagacattgttcaatctcttagtcg
sgRm-F2b	gtttcagagctatgctggaaacagc
Assembly of final CRISPR/Cas9 binary vectors (5'→3')	
oETC2-F	attggaagtgagtagcatcgaat
oETC2-R	aaacattcgatgctactcacttc
oBRI1-F	attgtgggtcataacgatatctc
oBRI1-R	aaacgagatatcgttatgaccca
oT&C-F	attgaatatctctctatctcctc

oT&C-R	aaacgaggagatagagagatatt
oETC2-F2	tgcaagaagtgagtagcatcgaat
oETC2-R2	aaacattcgatgctactcacttct
oBRI1-F2	tgcattgggtcataacgatatctc
oBRI1-R2	aaacgagatatcgttatgacccaa
oT&C-F2	tgcacaatatctctctatctcctc
oT&C-R2	aaacgaggagatagagagatattg
oADH1-F	attgtatcttcggccatgaagctg
oADH1-R	aaaccagcttcatggccgaagata
T1-BsF@T&C	ataacattggtctcatgcagaatatctctctatctcctc
T1sgR-F0@T&C	aatatctctctatctcctcgttttagagctagaaatagc
T2tRM-R0@E	acattcgatgctactcacttcctatcagagccaggtttcg
T2-BsR@E	atacattatggtctcgaaacattcgatgctactcacttc
T1-BsF@T&C2	ataacattggtctcatgcacaatatctctctatctcctc
T2tRM-R0@E2	acattcgatgctactcacttcttatcagagccaggtttcg
T1-BsF@E	ataacattggtctcatgcaggaagtgagtagcatcgaat
T1sgR-F0@E	gaagtgagtagcatcgaatgttttagagctagaaatagc
T2tRM-R0@T&C	acgaggagatagagagatattctatcagagccaggtttcg
T2-BsR@T&C	atacattatggtctcgaaacgaggagatagagagatatt
T1-BsF@E2	ataacattggtctcatgcaagaagtgagtagcatcgaat
T2tRM-R0@T&C2	acgaggagatagagagatattgtatcagagccaggtttcg
T2tRG-R0@E2	acattcgatgctactcacttcttgcaccagccgggaatc
T1-sgRm-F0@T&C	caatatctctctatctcctcgtttcagagctatgctggaaacagc
Generation of transgenic Arabidopsis plants and analysis of mutations and T-DNA insertions (5'→3')	
TRY-FP	ttcgctctataaactctcatctctcacg
TRY-RP	acctaaccgcatggattaaagttgattacc
CPC-FP	gtttcgtgtcttcagattagttcgatgt
CPC-RP	gagctacctcgttgacccatatcgt
ETC2-F	cagtagttatggataataccaaccgtct
ETC2-R	atcagctttgatttgttactctcgccat
5G50230-F	cacggataaggtttgtgctgtggat
5G50230-R	ccgtttttatcagccgatgccaatg
BRI1-F	aacgatgggatgaagaaagagtgtc
BRI1-R	tgtccttccgcatacatctccaact
ADH1-F	tgatcggttacatttctgttgaggt
ADH1-R	cgcctaaaccaacagcaccaagacc
35St-F0	cataataatgtgtgagtagttcccagat
35St-F	agggttcctatagggtttcgctcat
Lac-F0	ccaacttaatcgccttgcagcacat
Lac-F	ttcccaacagttgcgcagcctgaat

Table S8

Table S8 Features of cloning CRISPR/Cas9 binary vectors

Vector name	sgRNA cassette	Cas9 promoter	Cas9 variant	mCherry
pHEE401	U6-BB-sgR	EC1.2p	zCas9	/
pHEE401E	U6-BB-sgR	EC1fp	zCas9	/
pHME1	U6-BB-sgR	EC1fp	zCas9	Yes
pHME2	U6-BB-sgRm	EC1fp	zCas9	Yes
pHME21	U6-BB-sgRm	EC1fp	zCas9dF	Yes
pHME3	U6-tRNA-BB-sgRm	EC1fp	zCas9	Yes
pHME31	U6-tRNA-BB-sgRm	EC1fp	zCas9dF	Yes
pHE2	U6-tRNA-BB-sgR	EC1fp	zCas9	/
pHE3	U6-tRNA-BB-sgRm	EC1fp	zCas9	/
pHE31–38	U6-tRNA-BB-sgRm	EC1fp	8 variants	/
pNNGRRT2	U6-BB-sgR@Sa	EC1fp	SaCas9	/

For pHME: H, hygromycin resistance; M, mCherry; E, egg cell-specific promoter controlled CRISPR/Cas9 system. For sgR/sgRm, original/mutant sgRNA scaffold. For BB, BsaI–BsaI sites of multiple cloning sites for assembly of sgRNA cassettes. For sgR@Sa, SaCas9-specific sgRNA scaffold. zCas9, *Zea mays* codon-optimized SpCas9. 8 variants, 8 high-specificity zCas9 variants.

Table S9

Table S9 Structures of the PCR templates used in the assembly of sgRNA cassettes

Vector name	Structure of PCR template	Structure of PCR fragment
pCBC-sgRtRM	sgR-tRNA_Met	BsaI-T1-//-T2-BsaI
pCBC-sgRtRG	sgR-tRNA_Gly	BsaI-T1-//-T2-BsaI
pCBC-DT1T2	sgR-8×T-U6_29p	BsaI-T1-//-T2-BsaI
pCBC-DT1T2b	sgR-8×T-U6_29p	BsaI-T1-//-T2-BsaI
pCBC-DT1T2c	sgRm-8×T-U6_29p	BsaI-T1-//-T2-BsaI
pCBC-DT1T2d	sgR-8×T-U6_29p-tRNA_Met	BsaI-T1-//-T2-BsaI
pCBC-DT1T2e	sgRm-8×T-U6_29p-tRNA_Met	BsaI-T1-//-T2-BsaI

"//" represents the corresponding structure of the PCR template. The 8×T-containing U6-26t in pCBC-DT1T2b/c/d/e was shortened from 192 bp to 62 bp.

Table S10

Table S10 Final CRISPR/Cas9 binary vectors, each harboring one sgRNA cassette

Final vector	Cloning vector	Two oligos annealed
pEa/pEb	pHEE401/pHEE401E	oETC2-F + oETC2-R
pBa/pBb	pHEE401/pHEE401E	oBRI1-F + oBRI1-R
pEc–f	pHE2/pHE3/pHE32/pHE34	oETC2-F2 + oETC2-R2
pBc–f	pHE2/pHE3/pHE32/pHE34	oBRI1-F2 + oBRI1-R2
pT&Ca–e	pHME1/pHME2/pHME21/ pHME3/pHME31	oT&C-F + oT&C-R
pT&Cf, pT&Cg–n	pHE3, pHE31–38	oT&C-F2 + oT&C-R2
pA@Sa	pNNGRRT2	oADH1-F + oADH1-R

Cloning CRISPR/Cas9 binary vector and two oligos used to generate a final CRISPR/Cas9 binary vector are also listed.

Table S11

Table S11 Final CRISPR/Cas9 binary vectors each harboring two sgRNA cassettes

Final vector	Cloning vector	Primer mixture	PCR template
pT&C-E	pHE2	T1-BsF@T&C + T1sgR-F0@T&C + T2tRM-R0@E + T2-BsR@E	pCBC-sgRtRM
pT&C-E2	pHE2	T1-BsF@T&C2 + T1sgR-F0@T&C + T2tRM-R0@E2 + T2-BsR@E	pCBC-sgRtRM
pE-T&C	pHE2	T1-BsF@E + T1sgR-F0@E + T2tRM-R0@T&C + T2-BsR@T&C	pCBC-sgRtRM
pE-T&C2	pHE2	T1-BsF@E2 + T1sgR-F0@E + T2tRM-R0@T&C2 + T2-BsR@T&C	pCBC-sgRtRM
pT&C-E2b	pHE2	T1-BsF@T&C2 + T1sgR-F0@T&C + T2tRG-R0@E2 + T2-BsR@E	pCBC-sgRtRG
pT&C-E3a	pHE2	= T&C-E2 primer Mixture	pCBC-DT1T2d
pT&C-E3b	pHE3	T1-BsF@T&C2 + T1sgRm-F0@T&C + T2tRM-R0@E2 + T2-BsR@E	pCBC-DT1T2e

Cloning CRISPR/Cas9 binary vector, primer mixture, and PCR template used to generate a final CRISPR/Cas9 binary vector are also listed.

Methods S1

Vector construction

All primers used in this study are listed in Table S7. The cloning CRISPR/Cas9 binary vectors, the PCR template vectors, the final CRISPR/Cas9 binary vectors each harboring one sgRNA cassette, and the final CRISPR/Cas9 binary vectors, each harboring two sgRNA cassettes, are listed in Tables S8–S11, respectively. Annotated sequences of the sgRNA cassettes for cloning are provided in Appendix S1.

Generation of sgRNA cassettes and Cas9 variants

We amplified the U6 cassette with primers U6-BNF/-BsR from pHEE401E (Wang et al. 2015) by PCR, and cloned the purified PCR fragment into pCBC, which generated pCBC-U6SsgR. We amplified the ampicillin-resistance gene with primers Ap-BBF/BBR from pVLC-LBHU6A, digested the PCR fragment with *Bsa*I, and used the digested fragment to replace the *Bsa*I fragment (spectinomycin-resistance gene), resulting in production of pCBC-U6AsgR. We then introduced mutations in sgRNA scaffold by PCR amplification of the full-length pCBC-U6AsgR with primers sgRm-F/-SaR. We purified the PCR fragment, digested it with *Sac*I, blunted it with T4 DNA polymerase, and allowed it to self-ligate, resulting in production of pCBC-U6AsgRm. To obtain the tRNA-Gly gene, we amplified tRNA-Gly (AT1G06880) from Arabidopsis DNA with primers tRG-BbF/-R0/-BbR, cloned the purified PCR fragment into pCBC, generating pCBC-tRG. We amplified the *ApR* fragment from pCBC-U6AsgR with primers Ap-BBF/-BBR2, digested the purified fragment with *Bbs*I, and inserted it between two *Bsa*I sites of pCBC-tRG, thus producing pCBC-tRGA. We used the *Bbs*I tRNA-*ApR* containing fragment of pCBC-tRGA to replace the *ApR Bsa*I fragments of pCBC-U6AsgR and pCBC-U6AsgRm, thereby generating pCBC-U6tRAsgR and pCBC-U6tRAsgRm, respectively.

We subcloned the 1.0-kb *Xma*I-*Psh*AI fragment of zCas9 into pCBC and introduced point mutations into the fragment, thereby yielding mutant fragments e1.0, e1.1, e1.2, HF1b-e1.1, HF1b-e1.2, and HF1b. The e1.0 fragment harbored the K810A, K1003A, and R1060A mutations, the e1.1 fragment harbored the K848A, K1003A, and R1060A mutations, the e1.2 fragment harbored the K810A, K848A, K1003A, and R1060A mutations, the HF1b-e1.1 fragment harbored the K848A, Q926A, K1003A, and R1060A mutations, the HF1b-e1.2 fragment harbored the K810A, K848A, Q926A, K1003A, and R1060A mutations, and the HF1b fragment harbored the Q926A mutation. We used the six *Xma*I-*Psh*AI mutant fragments to replace the wild-

type *Xma*I-*Psh*AI fragment of pUC57-zCas9, thereby generating pUC57-zCas9(1.0)/(1.1)/(1.2), pUC57-HF1b-e(1.1)/-e(1.2), and pUC57-HF1b, respectively.

We subcloned the 0.8-kb *Nhe*I-*Xma*I fragment of zCas9 into pCBC and introduced point mutations into the fragment, resulting in production of a HF1a mutant fragment harboring N497A, R661A, and Q695A mutations. We used the *Nhe*I-*Xma*I mutant fragment to replace the wild-type *Nhe*I-*Xma*I fragments of pUC57-HF1b, pUC57-HF1b-e(1.1), pUC57-eCas9(1.1), and pUC57-eCas9(1.2), thereby producing pUC57-Cas9-HF1, pUC57-HF1-e(1.1), pUC57-HF1a-e(1.1), and pUC57-HF1a-e(1.2), respectively. We generated a 363-bp PCR fragment from zCas9 using primer pair zCas9dF-F/-R, which was then digested with *Bst*XI. The digested fragment was used to replace the *Bst*XI fragment of pUC57-zCas9, resulting in production of pUC57-zCas9dF. The codons encoding original/mutated amino acids are as follows: AAT/GCG (N497A), AGG/GCC (R661A), CAG/GCG (Q695A), AAG/GCG (K810A), AAG/GCC (K848A), CAG/GCC (Q926A), AAG/GCC (K1003A), and CGC/GCT (R1060A).

Construction of cloning CRISPR/Cas9 binary vectors

Some features of the cloning CRISPR/Cas9 binary vectors are summarized in Table S8. We generated a PCR fragment of the mCherry cassette from pHDE 35S Cas9-mCherry (Gao et al. 2016) with primer pair mCherry-HMF/-EAR and cloned it into pCBC, thereby generating pCBC-mCh. We disrupted the SacI site in the mCherry cassette, producing pCBC-mChe-dSa. We generated the mCherry fragment by digestion of pCBC-mChe-Sa with AvrII and EcoRI, and used the fragment to replace the sgRNA cassette of pNGG (Zhang et al. 2017), which was derived from pHEE401E (Wang et al. 2015), thereby yielding pHME0. We inserted the three sgRNA cassettes from pCBC-U6AsgR, pCBC-U6AsgRm, and pCBC-U6tRAsgRm into the NcoI and SpeI sites of pHME0, thereby producing pHME1, pHME2, and pHME3, respectively. We replaced zCas9 of pHME2 and pHME3 with zCas9dF of pUC57-zCas9dF, thereby generating pHME21 and pHME31, respectively.

We inserted the two sgRNA cassettes from pCBC-U6tRAsgR and pCBC-U6tRAsgRm into the *Nco*I and *Spe*I sites of pNGG (Zhang et al. 2017), thereby producing pHE2 and pHE3, respectively. The pHEE401E can be regarded as pHE1. We replaced *zCas9* of pHE3 with *eCas9(1.0)*, *eCas9(1.1)*, *eCas9(1.2)*, *Cas9-HF1*, *HF1-e(1.1)*, *HF1a-e(1.1)*, *HF1b-e(1.2)*, and *HF1a-e(1.2)* cloned in pUC57, thereby generating pHE31–38, respectively.

We used a short insert generated by annealing two oligos oAiCE-F/-R to replace the sgRNA cassette of

pNNGRRT (Zhang et al. 2017), yielding pNNGRRT0. We amplified the sgRNA cassette with primers U6-BNF/-BsR from pNNGRRT, digested the purified fragment with *Nco*I and *Spe*I, which were then inserted into the *Nco*I and *Spe*I sites of pNNGRRT0, thereby producing pNNGRRT2.

Generation of PCR template vectors

Structures of the PCR templates for the assembly of two sgRNA cassettes are summarized in Table S9. We amplified the *SpR*-sgR fragment with primers Sp-XhF/sgR-BbR from pHEE401E, and the tRNA_Gly-*ApR* fragment with primers tRG-BbF/Ap-XhR. The two fragments were mixed, purified, digested with *Bbs*I, and ligated with pCBC, yielding pCBC-sgRtRG. We amplified the *SpR*-sgR fragment with primers Sp-XhF/sgR-BbR from pHEE401E (Wang et al. 2015), and the tRNA_Met fragment with primers tRM-BbF/-R. The two fragments were mixed, purified, digested with *Bbs*I, and ligated with pCBC, thereby generating pCBC-sgRtRM.

We shortened the 8×T-containing U6-26t of pCBC-DT1T2 from 192 bp to 62 bp, thereby generating pCBC-DT1T2b. We introduced mutations into the sgRNA scaffold of pCBC-DT1T2b by PCR amplification with primers sgRm-F/-F0/U629-R. We purified the PCR fragment, and ligated it with pCBC, thereby producing pCBC-DT1T2c. We amplified the sgR-8×T-U6_29p fragment with primers sgR-F/U6_29-BbR from pCBC-DT1T2b, and the tRNA_Met fragment with primers tRM-BbF/-R. The two fragments were then mixed, purified, digested with *Bbs*I, and ligated with pCBC, yielding pCBC-DT1T2d. We amplified the sgRm-8×T-U6_29p fragment with primers sgRm-F2b/U629-BbR from pCBC-DT1T2c, and the tRNA_Met fragment with primers tRM-BbF/-R. The two fragments were mixed, purified, digested with *Bbs*I, and ligated with pCBC, thereby producing pCBC-DT1T2e.

Assembly of the final CRISPR/Cas9 binary vectors

The final CRISPR/Cas9 binary vectors, together with the cloning CRISPR/Cas9 binary vectors, oligos, or primers, and PCR template vectors are summarized in Tables S10 and S11.

To generate CRISPR/Cas9 binary vectors with each harboring one sgRNA cassette, we inserted a target sequence formed by annealing two oligos into a cloning binary vector linearized with *Bsa*I digestion, thereby producing a final CRISPR/Cas9 vector harboring one sgRNA. To generate CRISPR/Cas9 binary vectors with each harboring two sgRNA cassettes, we generated a PCR fragment using four primers, which was then purified, digested with *Bsa*I, and ligated with a cloning binary vector linearized with *Bsa*I digestion, thereby

yielding a final CRISPR/Cas9 vector harboring two sgRNA.

References

Gao X, Chen J, Dai X, Zhang D, Zhao Y (2016) An effective strategy for reliably isolating heritable and Cas9-free Arabidopsis mutants generated by CRISPR/Cas9-mediated genome editing. Plant Physiol 171:1794-1800

Wang ZP, Xing HL, Dong L, Zhang HY, Han CY, Wang XC, Chen QJ (2015) Egg cell-specific promoter-controlled CRISPR/Cas9 efficiently generates homozygous mutants for multiple target genes in Arabidopsis in a single generation. Genome Biol 16:144

Zhang HY, Wang XH, Dong L, Wang ZP, Liu B, Lv J, Xing HL, Han CY, Wang XC, Chen QJ (2017) MISSA 2.0: an updated synthetic biology toolbox for assembly of orthogonal CRISPR/Cas systems. Sci Rep 7:41993

Appendix S1–S5

Appendix S1. Sequence of the U6-tRNA-BB-sgRm cassette for cloning

(U6-26p)-(tRNA-Gly)-(BsaI-ApR-BsaI)-sgRm-(U6-26t)

CGACTTGCCTTCCGCACAATACATCATTTCTTCTTAGCTTTTTTTCTTCTTCTTCGTTCATACAGTTTTTTTTTGTTTATCAGC
TTACATTTTCTTGAACCGTAGCTTTCGTTTTCTTCTTTTTAACTTTCCATTCGGAGTTTTTGTATCTTGTTTCATAGTTTGTCC
CAGGATTAGAATGATTAGGCATCGAACCTTCAAGAATTTGATTGAATAAAACATCTTCATTCTTAAGATATGAAGATAATCT
TCAAAAGGCCCCTGGGAATCTGAAAGAAGAGAAGCAGGCCCATTTATATGGGAAAGAACAATAGTATTTCTTATATAGGC
CCATTTAAGTTGAAAACAATCTTCAAAAGTCCCACATCGCTTAGATAAGAAAACGAAGCTGAGTTTATATACAGCTAGAGT
CGAAGTAGTGATTGAACAAAGCACCAGTGGTCTAGTGGTAGAATAGTACCCTGCCACGGTACAGACCCGGGTTCGATTC
CCGGCTGG**TGCA**TGAGACCTTCGGGGAAATGTGCGCGGAACCCCTATTTGTTTATTTTTCTAAATACATTCAAATATGTATCCGCTCATGGGACAATAACCCTGATAAATGCTTCAATAATATTGAAAAAGGAAGAGTATG
AGTATTCAACATTTCCGTGTCGCCCTTATTCCCTTTTTTGCGGCATTTTGCCTTCCTGTTTTTGCTCACCCAGAAACGCTGGTGAAAGTAAAAGATGCTGAAGATCAGTTGGGTGCACGAGTGGGTTACATCGAACTGGATCTCAACAGCGGTAAGATCCTTGAGAGTTTTCGCCCC
GAAGAACGTTTTCCAATGATGAGCACTTTTAAAGTTCTGCTATGTGGCGCGGTATTATCCCGTATTGACGCCGGGCAAGAGCAACTCGGTCGCCGCATACACTATTCTCAGAATGACTTGGTTGAGTACTCACCAGTCACAGAAAAGCATCTTACGGATGGCATGACAGTAAGAGA
ATTATGCAGTGCTGCCATAACCATGAGTGATAACACTGCGGCCAACTTACTTCTGACAACGATCGGAGGACCGAAGGAGCTAACCGCTTTTTTGCACAACATGGGGGATCATGTAACTCGCCTTGATCGTTGGGAACCGGAGCTGAATGAAGCCATACCAAACGACGAGCGTGAC
ACCACGATGCCTGTAGCAATGGCAACAACGTTGCGCAAACTATTAACTGGCGAACTACTTACTCTAGCTTCCCGGCAACAATTAATAGACTGGATGGAGGCGGATAAAGTTGCAGGACCACTTCTGCGCTCGGCCCTTCCGGCTGGCTGGTTTATTGCTGATAAATCTGGAGCCGG
TGAGCGTGGCTCTCGCGGTATCATTGCAGCACTGGGGCCAGATGGTAAGCCCTCCCGTATCGTAGTTATCTACACCACGGGGAGTCAGGCAACTATGGATGAACGAAATAGACAGATCGCTGAGATAGGTGCCTCACTGATTAAGCATTGGTAACTGTCAGACCAAGTTTACTCAT
ATATACTTTAGATTGATTTAAAACTTCATTTTTAATTTAAAAGGATCTAGGTGAAGATCCTTTTTGATAATCCCATGACCAAAATCCCTTAACGTGAGTTTTCGTTCCACTGAGCGTCAGGTCTCA**GTTT**CAGAGCTATG
CTGGAAACAGCATAGCAAGTTGAAATAAGGCTAGTCCGTTATCAACTTGAAAAAGTGGCACCGAGTCGGTGCTTTTTTT
TGCAAAATTTTCCAGATCGATTTCTTCTTCCTCTGTTCTTCGGCGTTCAATTTCTGGGGTTTTCTCTTCGTTTTCTGTAACTG
AAACCTAAAATTTGACCTAAAAAAAAATCTCAAATAATATGATTCAGTGGTTTTGTACTTTTCAGTTAGTTGAGTTTTGCAGT
TCCGATGAGATAAACCAATA

Notes:

1. ApR, ampicillin-resistance gene; sgRm, mutant sgRNA scaffold. The enlarged letters indicate *Bsa*I sites, whereas the underlined letters indicate positions of 5' sticky ends produced by digestion with *Bsa*I.
2. The only difference between the U6-tRNA-BB-sgR cassette and the U6-tRNA-BB-sgRm cassette is that the original rather than mutant sgRNA scaffold was used.
3. To generate a final CRISPR/Cas9 binary vector harboring one or two sgRNA cassettes, the *Bsa*I-*ApR*-*Bsa*I fragment was replaced by a short insert produced by annealing two 24-nt oligos harboring a target sequence or a PCR fragment harboring two target sequences.

Appendix S2. Sequence of the PCR fragment amplified from pCBC-sgRtRM

BsaI-T1-sgR-(tRNA-Met)-T2-BsaI

GGTCTCA**TGCA**NNNNNNNNNNNNNNNNNNNNGTTTTAGAGCTAGAAATAGCAAGTTAAAATAAGGCTAGT
CCGTTATCAACTTGAAAAAGTGGCACCGAGTCGGTGCAACAACAAATCAGAGTGGCGCAGCGGAAGCGTGGTGGGCC
CATAACCCACAGGTCCCAGGATCGAAACCTGGCTCTGATANNNNNNNNNNNNNNNNNNNN**GTTT**CGAGAC
C

Notes:

1. T1/T2, Target sequence-1/2; sgR, sgRNA scaffold. The enlarged letters indicate *Bsa*I sites, whereas the underlined letters indicate positions of the 5' sticky ends that were generated by digestion with *Bsa*I.

Appendix S3. Sequence of the PCR fragment amplified from pCBC-sgRtRG

BsaI-T1-sgR-(tRNA-Gly)-T2-BsaI

GGTCTCA**TGCA**NNNNNNNNNNNNNNNNNNNNGTTTTAGAGCTAGAAATAGCAAGTTAAAATAAGGCTAGT
CCGTTATCAACTTGAAAAAGTGGCACCGAGTCGGTGCAACAAAGCACCAGTGGTCTAGTGGTAGAATAGTACCCTGCCA
CGGTACAGACCCGGGTTCGATTCCCGGCTGGTGCANNNNNNNNNNNNNNNNNNNN**GTTT**CGAGACC

Notes:

1. T1/T2, Target sequence-1/2; sgR, sgRNA scaffold. The enlarged letters indicate *Bsa*I sites, whereas the underlined letters indicate positions of the 5' sticky ends that were generated by digestion with *Bsa*I.

Appendix S4. Sequence of the PCR fragment amplified from pCBC-DT1T2d

BsaI-T1-sgR-(U6-26t)-(U6-29p)-(tRNA-Met)-T2-BsaI

GGTCTCA**TGCA**NNNNNNNNNNNNNNNNNNNNGTTTTAGAGCTAGAAATAGCAAGTTAAAATAAGGCTAGT
CCGTTATCAACTTGAAAAAGTGGCACCGAGTCGGTGCTTTTTTTTGCAAAATTTTCCAGATCGATTTCTTCTTCCTCTGTT
CTTCGGCGTTCAATTTCTTTAATCCAAACTACTGCAGCCTGACAGACAAATGAGGATGCAAACAATTTTAAAGTTTATCT
AACGCTAGCTGTTTTGTTTCTTCTCTCTGGTGCACCAACGACGGCGTTTTCTCAATCATAAAGAGGCTTGTTTTACTTAA
GGCCAATAATGTTGATGGATCGAAAGAAGAGGGCTTTTAATAAACGAGCCCGTTTAAGCTGTAAACGATGTCAAAAA
CATCCCACATCGTTCAGTTGAAAATAGAAGCTCTGTTTATATATTGGTAGAGTCGACTAAGAGATTGAACAACAAATCA
GAGTGGCGCAGCGGAAGCGTGGTGGGCCCATAACCCACAGGTCCCAGGATCGAAACCTGGCTCTGATANNNNNNNN
NNNNNNNNNNNN**GTTT**CGAGACC

Notes:

1. T1/T2, Target sequence-1/2; sgR, sgRNA scaffold. The enlarged letters indicate *Bsa*I sites, whereas the underlined letters indicate positions of the 5' sticky ends that were generated by digestion with *Bsa*I.

Appendix S5. Sequence of the PCR fragment amplified from pCBC-DT1T2e

BsaI-T1-sgRm-(U6-26t)-(U6-29p)-(tRNA-Met)-T2-BsaI

GGTCTCA**TGCA**NNNNNNNNNNNNNNNNNNNNGTTTCAGAGCTATGCTGGAAACAGCATAGCAAGTTGAA
ATAAGGCTAGTCCGTTATCAACTTGAAAAAGTGGCACCGAGTCGGTGCTTTTTTTTGCAAAATTTTCCAGATCGATTTCTT
CTTCCTCTGTTCTTCGGCGTTCAATTTCTTTAATCCAAACTACTGCAGCCTGACAGACAAATGAGGATGCAAACAATTTTA
AAGTTTATCTAACGCTAGCTGTTTTGTTTCTTCTCTCTGGTGCACCAACGACGGCGTTTTCTCAATCATAAAGAGGCTTG
TTTTACTTAAGGCCAATAATGTTGATGGATCGAAAGAAGAGGGCTTTTAATAAACGAGCCCGTTTAAGCTGTAAACGA
TGTCAAAAACATCCCACATCGTTCAGTTGAAAATAGAAGCTCTGTTTATATATTGGTAGAGTCGACTAAGAGATTGAAC
AACAAATCAGAGTGGCGCAGCGGAAGCGTGGTGGGCCCATAACCCACAGGTCCCAGGATCGAAACCTGGCTCTGATAN
NNNNNNNNNNNNNNNNNNN**GTTT**CGAGACC

Notes:

1. T1/T2, Target sequence-1/2; sgRm, mutant sgRNA scaffold. The enlarged letters indicate *Bsa*I sites, whereas the underlined letters indicate positions of the 5' sticky ends that were generated by digestion with *Bsa*I.
